# The Microstructure and Mechanical Properties of a 15-6 PH Stainless Steel with Improved Thermal Aging Embrittlement Resistance

**DOI:** 10.3390/ma17051179

**Published:** 2024-03-03

**Authors:** Runtao Lv, Chenxin Yin, Bing Bai, Wen Yang, Zhangjian Zhou

**Affiliations:** 1 School of Materials Science and Engineering, University of Science and Technology Beijing, Beijing 100083, China; haoqizhouzhang@163.com (R.L.); m202210394@xs.ustb.edu.cn (C.Y.); 2 China Institute of Atomic Energy, Beijing 102413, China; yangwen@ciae.ac.cn

**Keywords:** martensite precipitation hardening stainless steel, Cu-rich phase, reversed austenite, thermal aging

## Abstract

The evolution of the microstructure and the mechanical properties of a 15-6 martensite precipitated hardened (15-6 PH) stainless steel after thermal treatment and long-term aging at 480 °C were investigated. Compared with 17-4 PH steel, the content of Cr decreased and Ni increased in the newly developed 15-6 PH steel; therefore, reversed austenite formed after thermal treatment at 620 °C of the solution-treated 15-6 PH steel. Although the reversed austenite may reduce the strength of the steel, it is very beneficial for the inhibition of the aging brittleness of the steel. During the accelerated thermal aging at 480 °C, the Cu-rich phase gradually coarsened, and its crystal structure changed, while the reversed austenite phase sightly increased and the Charpy impact energy maintained a rather high value. The increase of the reversed austenite content can offset the reduction of the strengthening effect of the Cu-rich phase and therefore maintain an excellent impact property of the material after thermal aging.

## 1. Introduction

Due to its good welding performance, high mechanical strength, and good corrosion resistance, martensitic precipitation hardening stainless steel with low carbon content, particularly Cu-added 17-4 PH stainless steel and 15-5 PH stainless steel, has been extensively employed in harsh environments, such as in the petroleum, chemical, and nuclear energy industries [1,2,3,4,5,6,7,8]. As a kind of typical precipitation alloy, this material can be hardened by fine precipitates with a high number density after a series of thermal treatments, including high temperature solution treatment (usually at 1040 °C for 1–4 h), then multi-stage aging at temperatures between 450 °C and 620 °C for several hours. The nano-scaled Cu-rich phase is the most common strengthening phase formed throughout the aging process in the result of the precipitation of supersaturated Cufrom the matrix [9]. Carbides (mostly NbC) may also form during aging based on composition design [9,10,11,12,13]. However, it was reported that after years of service using it as valves in nuclear power plants at temperatures between 250–300 °C or after an accelerated thermal aging test at 350–500 °C, 17-4 PH stainless steel exhibited obvious thermal aging embrittlement, which means an increase in hardness and strength, but an obvious decrease in elongation and Charpy impact energy [9,14,15,16]. This is very harmful to the safe service of components. The evolution of the Cu-rich phase as well as the Cr-rich phase (α′ phase) formed by spinodal decomposition during long-term aging was considered the primary cause of this problem [9,15,17].

Numerous research works have indicated that throughout the aging process, the Cu-rich phase’s crystal structure will be changed from BCC to 9R and then to FCC, and the shape will be gradually coarsened from spherical to ellipsoidal or rod-like [10,11,15,16,17,18,19,20,21,22]. In this process, Mn shows a significant effect on the coarsening of the Cu-rich phase, which is a common alloying element in 17-4 PH steel.

On the other hand, it is well known that the Fe–Cr binary alloys are vulnerable to embrittlement when aged at 300~525 °C [23], which is called “475 °C embrittlement” due to the redistribution of Cr and the formation of brittle Cr-rich phase. The Cr content is quite high in 17-4 PH steel, according to the Fe–Cr binary phase diagram [24], and the Cr concentration of 17-4 PH is in the miscibility gap at 480 °C. Therefore, the martensite matrix will separate into a Cr-rich α′ phase and Fe-rich α phase during aging. Nucleation growth and spinodal decomposition are the two processes that can lead to the emergence of the Cr-rich phase. Both have been reported for 17-4 PH [17,25,26,27]. The formation of the α′ phase may increase the strength but decrease the ductility of the steel, obviously.

Extensive research has been performed on the evolution of the precipitated phases of 17-4 PH during long-term aging. According to Yeli et al. [9], the precipitation sequence of 17-4 PH during 480 °C aging was as follows: CrN/NbN precipitates at dislocations and matrix defects → Cu-rich precipitates and Nb-rich precipitates → Cr-rich precipitates → G phase. Wang et al. [15] considered that the evolution of precipitates during aging at 450 °C is as follows: Cu-rich clusters → Cu-rich clusters with core-shell structure (Cu-rich core, Ni-Mn-Si-rich shell)/(Nb, Mo)-rich clusters → non-twinned 9R-Cu/Ni-Mn-Si-Nb-rich clusters/Cr-rich α′ phase → W-type 9R-Cu/Ni-Mn-Si-Nb-rich clusters/Cr-rich α′ phase → twinned 9R-Cu/BCC-NMSN (Ni, Mn, Si, Nb-rich G phase)/Cr-rich α′ phase. However, the reports on the changes in Charpy impact energy after thermal aging treatment are limited compared with the works on microstructure evolution. The obtained results agree that the evolution and coarsening of the Cu-rich phase, as well as the precipitation of the Cr-rich phase, accounted for the aging embrittlement, while if a reversed-austenite phase can be formed during the aging process, it will help to reduce the negative effects of aging embrittlement.

Both precipitates and the reversed-austenite phase are related to composition design. Therefore, X5CrNiCuMo15-06 was advised as the upgrade material of 17-4 PH in the RCC-M 2007 edition in France (Design and Construction Rules for Mechanical Components of PWR Nuclear Islands) [28] in order to improve the thermal aging embrittlement behavior of martensitic precipitation hardening stainless steel when in long-term service at temperatures higher than 250 °C. Compared with 17-4 PH, the content of Cr and Cu was decreased while the content of Ni was increased and Mo was added in X5CrNiCuMo15-06. However, some research has indicated that the Mo may encourage the creation of Cr-rich and G phases, which have a negative impact on performance [29,30]. Up until now, there is little research focused on X5CrNiCuMo15-06. In this study, based on the X5CrNiCuMo15-06 standard composition, a 15-6 PH material was designed by removing Mn and replacing Mo with W. The change in microstructure evolution and mechanical properties, including the tensile property and Charpy impact test, after thermal treatments, especially during long-term thermal aging at 480 °C, were investigated. The mechanism of hardening and embrittlement was explained in comparison to 17-4 PH.

## 2. Experimental Materials and Methods

**Figure 1:**
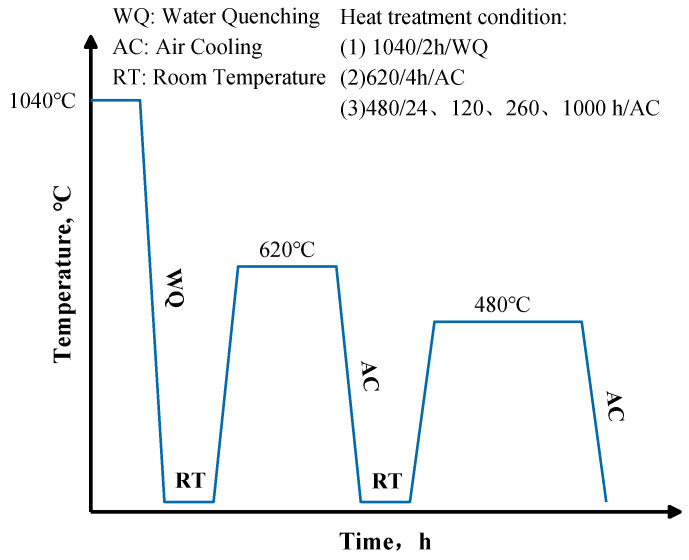
Flowchart for heat treatment process.

The investigated steel was melted by vacuum induction furnace, then treated by hot forging at temperature of 1100 °C with a forging ratio of 3:1. The actual composition of the material (designated 15-6 PH) as shown in Table 1 was measured by X–ray fluorescence spectrometry. A 17-4 PH steel was also fabricated for comparison. Its actual composition was also listed in Table 1. The heat treatment method utilized in this work was known as H1150. This heat treatment process involved solid solution treatment at 1040 °C for 2 h, which can dissolve the remaining precipitates of the as-received steel, followed by water quenching, then aging at 620 °C for 4 h, and air cooling to precipitate a large amount of strengthening phases in the martensite matrix, which improves the strength and hardness of the material. H1150 can provide the best combination of high strength, high hardness, and high toughness for 17-4 PH steel. It is one of the most common heat treatment processes for precipitation hardening martensitic steels specified in the standard, such as ASTM A693 [31]. Therefore, we also choose this heat treatment parameter for the newly developed 15-6 PH steel in this initial work for better understanding the effect of alloying elements content on the precipitation of Cu-rich strengthening phase. The heat-treated samples were then subjected to accelerated thermal aging for 24 h, 120 h, 260 h, and 1000 h, respectively, at 480 °C. Figure 1 depicts the flow chart for heat treatment process.

JMatPro software V7.0 was used for thermodynamic calculation of the experimental steel. The D/MAX-2500 X-ray diffractometer (XRD, Rigaku, Tokyo, Japan) was used to analyze the materials’ phases. Each step lasted for one second and had a step width of 0.1 degrees with a scanning range of 40° to 100°. The Tecnai F20 transmission electron microscope (TEM, FEI, Tokyo, Japan) was used to analyze the microstructure. Double spray thinning was used to prepare TEM samples. The voltage was 30 V, the temperature was −30 °C, and the electrolyte was a 10 vol% HClO_4_ methanol electrolyte.

Tensile specimens with a thread diameter of 6 mm were prepared perpendicular to the forging direction for the tensile test. Its original gauge length L_0_ = 15 mm, original diameter d_0_ = 3 mm. The tensile test has a strain rate of 10^−4^ s^−1^. The sample size for the Charpy impact test was 10 mm × 10 mm × 55 mm, and the test was conducted at 0 °C.

## 3. Results and Analysis

### 3.1. Thermodynamic Calculation and Phase Analysis

**Figure 2:**
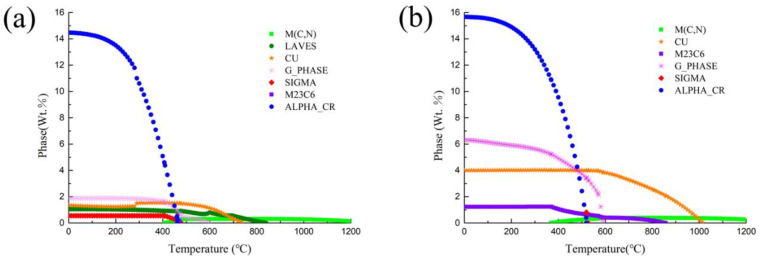
Thermodynamic calculation results: (**a**) 15-6 PH, (**b**) 17-4 PH.

Figure 2 shows the thermodynamic calculation results for the 15-6 PH and 17-4 PH steels. Both steels show precipitated phases including a Cr–rich phase, a Cu–rich phase, a G phase, and carbides such as MC and M_23_C_6_. But the amount of Cu precipitates and Cr-rich phases in 15-6 PH are significantly less than those in 17-4 PH, as the content of Cu and Cr is lower in 15-6 PH than in 17-4 PH. It should be noted that G phase was found in Figure 2. At present, there is still inconsistency in the views on the function of the G phase. Some studies suggest that the G phase can lead to aging embrittlement, while some reports believe that the G phase can be a beneficial strengthening phase. The exact conditions that led to its formation are still unclear. But it is believed that alloying elements such as Mn or Mo will promote the formation of the G phase. This is why the production of the G phase is considerably diminished in 15-6 PH due to the removal of Mn.

**Figure 3:**
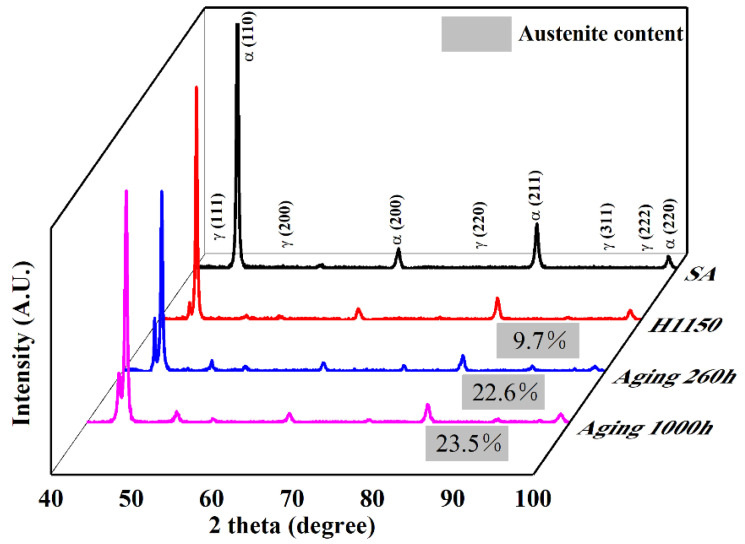
XRD patterns in different states.

Figure 3 shows the XRD diagram of 15-6 PH after various heat treatments. The material shows a single martensite phase after solution annealing (SA). Then, a tiny austenite peak can be discovered after H1150 heat treatment, indicating the development of reversed austenite (RA). The volume fraction of austenite was determined using the method proposed by Tanaka et al. [32]. The calculations revealed that the volume fraction of RA in H1150 sample is 9.7%, and then it increased to 22.6% and 23.5%, respectively, after accelerated thermal aging for 260 h and 1000 h at 480 °C.

### 3.2. Microstructure of 15-6 PH after H1150 Heat Treatment

**Figure 4:**
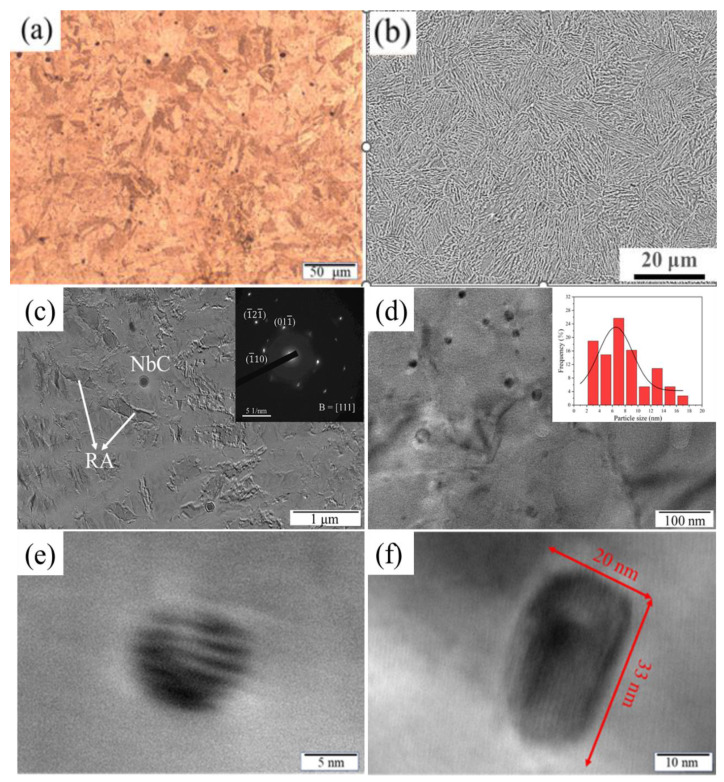
Metallographic and TEM photographs of 15-6 PH after heat treatment: (**a**,**b**): Martensite structure obtained after solid solution; (**c**): TEM image after H1150; (**d**): Nanoscale Cu-rich phase; (**e**): 9R-Cu Cu-rich phase; (**f**): FCC-Cu Cu-rich phase.

Figure 4a and Figure 4b are optical and SEM morphology images, respectively, which depict the full martensitic structure formed after solution treatment. Figure 4c shows the TEM picture of 15-6 PH after H1150 treatment. Apart from tempered martensite, a few RA grains and NbC precipitate particles with a size of roughly 150 nm can be found. This is different from 17-4 PH after similar heat treatment, in which only martensite can be found but which is absent of austenite [1,11]; however, it is similar to the behavior of a 15-5 PH which has a closer composition to the investigated 15-6 PH [13]. The reason why RA is more prone to form in 15-6 PH after heat treatment is due to its higher content of austenite-forming Ni but a lower content of ferrite-forming Cr compared with typical 17-4 PH. In fact, Hsiao et al. also reported that reversed austenite formed in a 17-4 PH after over-aging at 620 °C for 4 h [11]. It should point out that the Cr and Ni contents in their investigated steel are 15.7 and 4.89, respectively. The content of Cr is close to the bottom limit value of standard 17-4 PH, while the content of Ni is close to the upper limit value. Both the contents of Cr and Ni are quite close to those in 15-5 PH. This further indicates that running the same H1150 treatment may lead to an overheating of the austenite in 15-6 PH, thus leading to more RA at room temperature. It is necessary to optimize heat treatment parameters suitable for 15-6 PH steel.

The specific mechanism of the formation of RA and its relationship with composition design and procession parameters are still not very clear up until now. The occurrence of retained austenite after quenching within 15-5 PH alloy has been highlighted by Couturier et al. [13]. It is considered that both nucleation site and enrichment of austenite forming elements are important for the formation of reversed austenite. During the aging of martensitic steel, multiple precipitates, such as the Cu-rich phase will be precipitated at the boundary of martensitic flat noodles. They will modify the partitioning of solute elements that trigger austenite formation. Therefore, the precipitated Cu can serve as the heterogeneous nucleation site of reversed austenite in nickel-enriched regions and promote the growth of reversed austenite. The stability of reversed austenite will be influenced by element partitioning during the aging process.

At higher magnification observed by TEM, it can be seen that nanoscale Cu-rich phases are uniformly distributed in the matrix of 15-6 PH with a high number density after H1150 treatment, as shown in Figure 4d. The majority of the Cu-rich phases are spherical or oval with sizes of less than 20 nm. Here, the term Cu-rich phase was usually used for precipitation-hardened martensitic steels, as this phase can be tailed to change their crystal structure, as well as their size and shape, by micro-alloy composition design and post-heat treatments. In the investigated 15-6 PH steel, although most of the Cu-rich phase is 9R-Cu, FCC-Cu was also identified through a high-resolution TEM picture, as shown in Figure 4e and Figure 4f, respectively. The 9R-Cu phase in Figure 4e is spherical with a size of 10 nm, while the FCC-Cu is a rod shape with an axis–diameter ratio of 1.65; the long and short axes are 33 nm and 20 nm, respectively, as shown in Figure 4f. According to the statistics of several TEM pictures, the average size of Cu-rich precipitate is 7.86 nm with a number density of 2.50 × 10^22^ m^−3^. This number density is smaller than that reported in 17-4 PH after H1150 heat treatment [33], which is consistent with the thermodynamic calculation results shown in Figure 2, as the content of Cu in 15-6 PH is less than that in 17-4 PH.

### 3.3. Microstructure Evolution during Thermal Aging at 480 °C

**Figure 5:**
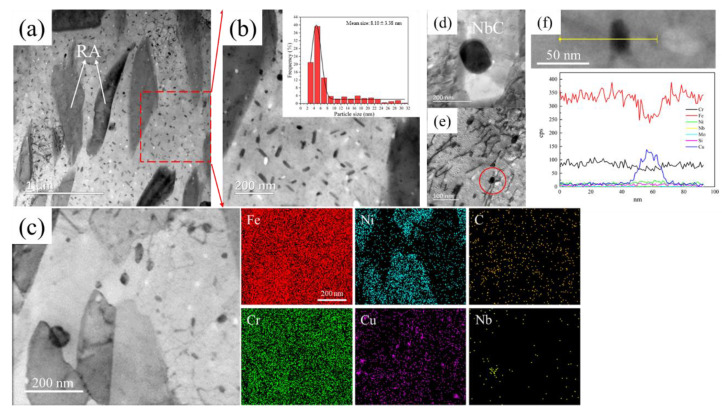
TEM and EDS spectra after accelerated thermal aging at 480 °C for 260 h: (**a**) TEM image of the matrix; (**b**) Cu-rich FCC structure; (**c**) EDS image; (**d**) NbC particle; (**e**) Dislocation bypasses the precipitated phase. (**f**) The EDS line scan of a Cu-rich particle. The red circle indicates the interaction between Cu-rich particle and dislocaiton. The yellow line indicates the EDS line scan range.

Figure 5 shows the TEM microstructure and EDS spectra of 15-6 PH after accelerated thermal aging at 480 °C for 260 h. Some long strip grains can be found in the matrix of martensite, as shown in Figure 5a,c, which was determined to be reversed austenite according to EDS analysis together with the XRD result in Figure 3. Figure 5a also shows numerous Cu-rich phases precipitated in the matrix. Higher magnification revealed that the Cu-rich phases are obviously coarsening during accelerated thermal aging at 480 °C compared with 15-6 PH after H1150 treatment. These evolved Cu-rich phases after the accelerated aging treatment featured a fundamental FCC crystal structure with an ellipsoidal and rod-like shape, according to Figure 5b.

Figure 5c shows the EDS map scanning of the RA which obviously enriched in Ni. In Figure 5c, there are also some Cu-rich phases with very fine particle sizes, which might be the result of the continuing precipitation of the Cu-rich phase during the accelerated thermal aging process at 480 °C. Previous works have shown that when 15-5 PH was tempered at 500 °C, the Cu-rich phase can continue to precipitate even after aging for 1000 h [18]. These fine Cu-rich phases have an average size of 8.10 nm and a number density of 4.53 × 10^22^ m^−3^. The precipitation of the Cu-rich phase and the diffusion segregation of Ni during accelerated thermal aging are related to the reverse transformation of martensite into austenite. The austenite phase is stabilized and the Ms in the immediate area is often reduced to below room temperature due to the segregation of Ni atoms during aging [34,35,36]. In addition, the formation of Cu-rich precipitates can further reduce the Mf temperature, resulting in the stability of austenite at room temperature [37]. As shown in Figure 5f, the line scan of a typical Cu-rich particle reveals that Ni is also slightly enriched in addition to the enrichment of Cu. This is because the interface energy between the Cu-rich phase and the matrix can be greatly reduced by the segregation of Ni.

The size of NbC essentially remained the same after the accelerated thermal aging process compared with the H1150 sample, as seen in Figure 5d. This result indicated that the NbC precipitate is much more stable than the Cu-rich precipitate during thermal aging. The possible reason is that the diffusion rate of Cu (5–6 × 10^−22^ m^2^/s) in α-Fe is much higher than that of Nb (5.4 × 10^−23^ m^2^/s); therefore, the Cu-rich phase is easy to nucleate in the matrix, and it is also easy to coarsen during thermal aging [9].

**Figure 6:**
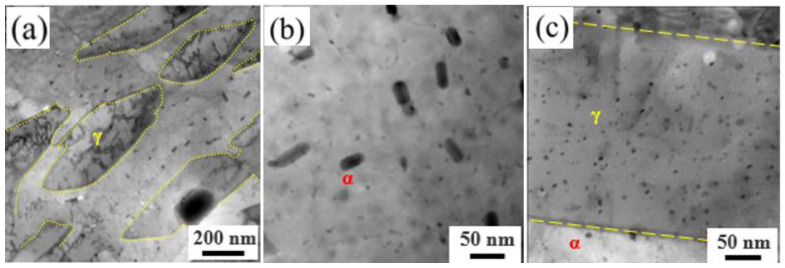
TEM pictures after accelerated thermal aging at 480 °C for 1000 h: (**a**) TEM image of the matrix, noted that the reversed austenite was circled by yellow line; (**b**) Cu-rich phase in martensite; (**c**) Cu-rich phase in reversed austenite.

Figure 6 shows the TEM microstructures of 15-6 PH after accelerated thermal aging at 480 °C for 1000 h. It is interesting to find that although coarsening occurred for some rod-like Cu-rich phases in martensite, there are also some fine Cu-rich phases continuously precipitated during the aging process, as shown in Figure 6b. Meanwhile, the Cu-rich phase in reversed austenite is still of very fine particle size, but the number density increased obviously after a longer aging time, as seen in Figure 6c. Therefore, the average size of Cu-rich particles decreased from 8.10 nm to 7.60 nm, while the number density increased from 4.53 × 10^22^ m^−3^ to 7.80 × 10^22^ m^−3^.

Figure 5e shows the relationship between dislocation and precipitated particles. It can be found that it is difficult for the dislocations to shear the fine precipitates (as indicated by the red circle), but they are pinned by particles. The rod-like Cu-rich phase with an FCC structure is incoherent with the matrix. As a result, it belongs to the eschewing the strengthening mechanism, and the Russell-Brown model is consistent with its strengthening contribution [17,38,39]:

(1) $$∆\tau \left(Cu\right)=\frac{\alpha Gb\sqrt{f_{Cu}}}{1.77<r>}$$

Here, $f_{Cu}$ is the volume fraction of the Cu-rich phase, and r is the size of the Cu-rich particle. Obviously, fine particle size and high number density of Cu-rich particles are beneficial for high strength. As mentioned above, there are both aging-coarsened Cu-rich particles and newly precipitated fine Cu-rich particles. Although the coarsening of Cu-rich particles during aging will have a negative influence on strength, the newly precipitated fine Cu-rich particles can compensate for it.

### 3.4. Changes of Mechanical Properties during Thermal Aging

**Figure 7:**
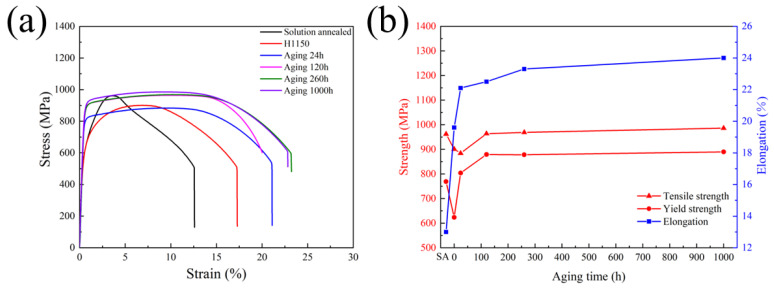
The effect of thermal treatments on tensile properties of 15-6 PH at room temperature. (**a**) The Stress-Strain curves. (**b**) The relationship between strength and elongation with the aging time.

According to previous works [10,11,40,41], the combined effect of tempering martensite, precipitates like the Cu-rich phase and Cr-rich phase, and the forming of reversed austenite will influence the evolution trend of strength and ductility of martensitic precipitation hardening stainless steel. Figure 7 shows the stress–strain curves and the relationship between yield strength and elongation with heat treatment conditions of the investigated steel, 15-6 PH. Both tensile strength and yield strength decreased after H1150 heat treatment compared with solid-solution-treated material, while its elongation is noticeably increased. This may be due to the formation of reversed austenite after heat treatment at 620 °C for 4 h, as indicated in Figure 4. During accelerated thermal aging at 480 °C, the strength increased first, then tended to stabilize. Although the Cu-rich particles formed after H1150 treatment will be coarsened during the aging process, fine Cu-rich particles continued to precipitate during the aging process as shown in Figure 5. These newly developed fine Cu-rich particles can obviously reinforce the matrix and keep their strength during the long-term aging process. It should be noted that the very fine α′ phase is also an important phase precipitated during the aging process of PH steels which may also counterbalance the softening effects of the coarsening of the Cu-rich phase and the formation of reversed austenite. Yeli et al. [9] evaluated the contribution of the relative strength of the α′ phase formed in 17-4 PH during aging at 480 °C. The results show that the α′ phase formed after aging for 24 h. With the extension of aging time, its contribution to strength can reach about 150 MPa. However, the size of the α′ phase is very fine and difficult to be characterized by TEM. APT analysis is necessary to further check and verify this.

**Figure 8:**
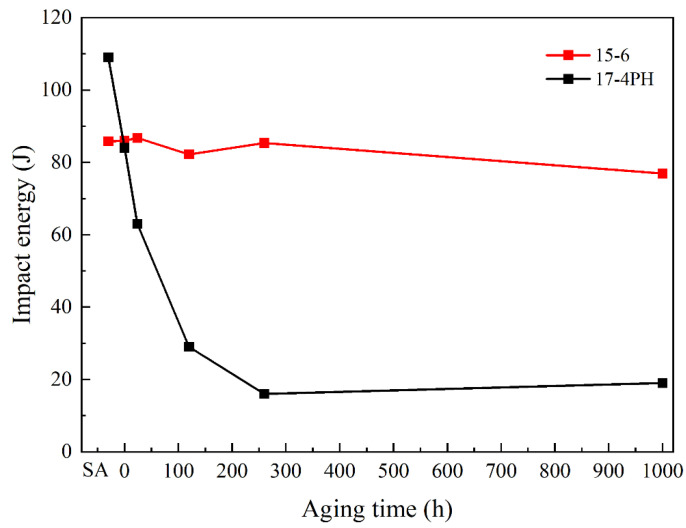
The relationship between thermal treatments and 0 °C impact energy.

Figure 8 shows the evolution of impact energy at 0 °C of the investigated material with different thermal treatment conditions; the result of 17-4 PH under the same conditions was also measured for comparison. For the solid-solution-annealed steels, the impact energy is higher than 80 J, and the impact energy of 17-4 PH is even higher than that of 15-6 PH. However, after the aging treatment, 17-4 PH shows a significantly rapid decline in impact energy, and then the impact energy only remained at less than 20 J as the aging time continued to extend. This result agrees with the reported works. However, for 15-6 PH material, the impact energy is surprisingly maintained at about 80 J, even after aging for 1000 h. This excellent performance is crucial for the long safe service of martensitic steels at high temperatures.

The unique aging brittleness resistance of 15-6 PH should be due to the fact that both the Cr and Cu contents of 15-6 PH are lower than those of 17-4 PH, while the Ni content is increased, which can decrease the negative effect of Cu-rich particle coarsening during the aging process. In particular, the composition design of 15-6 PH promoted the formation of reversed austenite during heat treatment, which is beneficial for the ductility of the material.

## 4. Conclusions

A 15-6 PH-based new grade martensitic steel with decreased Cr and Cu content and increased Ni content compared with 17-4 PH was designed and fabricated for potential applications in nuclear energy systems. The evolution of microstructure and mechanical properties during thermal treatment was investigated. The results can be summarized as follows:

(1) Reversed austenite formed after H1150 heat treatment in the 15-6 PH steel and its content increased following thermal aging at 480 °C.
(2) The Cu-rich precipitate coarsened and the crystal structure changed from the 9R to the FCC structure during thermal aging at 480 °C, while very fine Cu-rich particles precipitated in the reversed austenite during thermal aging at 480 °C.
(3) The tensile strength was quite stable after thermal aging at 480 °C for different lengths of time, while the elongation obviously increased compared with a solution-treated material.
(4) The 0 °C impact energy of 15-6 PH remained about 80 J after thermal aging at 480 °C up to 1000 h, which is nearly four times that of 17-4 PH. This demonstrated that the 15-6 PH material shows promising aging embrittlement resistance, while the procession and material cost are comparable with 17-4 PH. A longer aging time is necessary to evaluate the long-term performance of the steel in the near future.

## Figures and Tables

**Table 1:** The actual chemical composition of the experimental steel (wt.%).

Steel	Cr	Ni	Cu	W	Mn	Si	C	Nb	Fe
15-6 PH	15.04	5.99	1.54	0.66	0.06	0.30	0.03	0.38	Bal.
17-4 PH	17.20	3.87	3.52	-	0.85	0.43	0.05	0.21	Bal.

## Data Availability

The data that support the findings of this study are available upon reasonable request from the corresponding author (due to privacy).

## References

[1] Tavares S.S.M., Machado C.L.C., Oliveira I.G., Martins T.R.B., Masoumi M. (2017). Damage associated with the interaction between hydrogen and microstructure in a high sulfur 17-4PH steel for studs. Eng. Fail. Anal..

[2] Ping L., Cai Q.Z., Wei B.K., Zhang X.Z. (2006). Effect of Aging Temperature on Erosion-Corrosion Behavior of 17-4PH Stainless Steels in Dilute Sulphuric Acid Slurry. J. Iron Steel Res. Int..

[3] Raj S.V., Ghosn L.J., Lerch B.A., Hebsur M., Cosgriff L.M., Fedor J. (2007). Mechanical properties of 17-4PH stainless steel foam panels. Mater. Sci. Eng. A.

[4] Hara T., Semba H., Amaya H. (2022). Pipe and Tube Steels for Oil and Gas Industry and Thermal Power Plant. Encycl. Mater. Met. Alloys.

[5] Macha J.H., Kirby M.L., Hickey W.F., Riha D.S., Alston J.K., Holden B.B. (2021). Investigation of Thermal Embrittlement of 17-4PH Stainless Steel Main Steam Isolation Valves. J. Fail. Anal. Prev..

[6] Jin C., Zhou H., Lai Y., Li B., Zhang K., Chen H., Zhao J. (2021). Microstructure and mechanical properties of 15-5 PH stainless steel under different aging temperature. Met. Res. Technol..

[7] Brandl D., Lukas M., Stockinger M., Ploberger S., Ressel G. (2019). Evidence of austenite memory in PH 15-5 and assessment of its formation mechanism. Mater. Des..

[8] Wang K., Zhao J., Xie D., Lv F., Zhang Z., Wang H. (2022). Improved mechanical properties of laser-repaired 15-5PH stainless steel by in-situ heat treatment and grain refinement. Opt. Laser Technol..

[9] Yeli G., Auger M.A., Wilford K., Smith G.D., Bagot P.A., Moody M.P. (2017). Sequential nucleation of phases in a 17-4PH steel: Microstructural characterisation and mechanical properties. Acta Mater..

[10] Viswanathan U.K., Banerjee S., Krishnan R. (1988). Effects of aging on the microstructure of 17-4PH stainless steel. Mater. Sci. Eng. A.

[11] Hsiao C.N., Chiou C.S., Yang J.R. (2002). Aging reactions in a 17-4PH stainless steel. Mater. Chem. Phys..

[12] Habibi Bajguirani H.R. (2002). The effect of ageing upon the microstructure and mechanical properties of type 15-5PH stainless steel. Mater. Sci. Eng. A.

[13] Couturier L., De Geuser F., Descoins M., Deschamps A. (2016). Evolution of the microstructure of a 15-5PH martensitic stainless steel during precipitation hardening heat treatment. Mater. Des..

[14] Wang J., Zou H., Li C., Qiu S.Y., Shen B.L. (2006). The effect of microstructural evolution on hardening behavior of type 17-4PH stainless steel in long-term aging at 350 °C. Mater. Charact..

[15] Wang Z., Li H., Shen Q., Liu W., Wang Z. (2018). Nano-precipitates Evolution and Their Effects on Mechanical Properties of 17-4 Precipitation-hardening Stainless Steel. Acta Mater..

[16] Murayama M., Hono K., Katayama Y. (1999). Microstructural evolution in a 17-4 PH stainless steel after aging at 400 °C. Metall. Mater. Trans. A.

[17] Bai B., Hu R., Zhang C., Xue J., Yang W. (2021). Effect of precipitates on hardening of 17-4PH martensitic stainless steel serviced at 300 °C in nuclear power plant. Ann. Nucl. Energy.

[18] Zhou T., Babu R.P., Odqvist J., Yu H., Hedström P. (2018). Quantitative electron microscopy and physically based modelling of Cu precipitation in precipitation-hardening martensitic stainless steel 15-5 PH. Mater. Des..

[19] Lee T.H., Kim Y.O., Kim S.J. (2007). Crystallographic model for bcc-to-9R martensitic transformation of Cu precipitates in ferritic steel. Philos. Mag. A.

[20] Sun H., Li D., Diao Y., He Y., Yan L., Pang X., Gao K. (2022). Nanoscale Cu particle evolution and its impact on the mechanical properties and strengthening mechanism in precipitation-hardening stainless steel. Mater. Charact..

[21] Othen P.J., Jenkins M.L., Smith G.D.W., Phythian W.J. (1991). Transmission electron microscope investigations of the structure of copper precipitates in thermally-aged Fe-Cu and Fe-Cu-Ni. Philos. Mag. Lett..

[22] Othen P.J., Jenkins M.L., Smith G.D.W. (1994). High-resolution electron microscopy studies of the structure of Cu precipitates in α-Fe. Philos. Mag. A.

[23] Blackburn M.J., Nutting J. (1964). Metallography of an iron-21% chromium alloy subjected to 475 °C embrittlement. J. Iron Steel Inst..

[24] Shiao J.J., Tsai C.H., Kai J.J., Huang J.H. (1994). Aging embrittlement and lattice image analysis in a Fe–Cr–Ni duplex stainless steel aged at 400 °C. J. Nucl. Mater..

[25] Vitek J.M., David S.A., Alexander D.J., Keiser J.R., Nanstad R.K. (1991). Low temperature aging behavior of type 308 stainless steel weld metal. Acta Metall. Et. Mater..

[26] Hättestrand M., Larsson P., Chai G., Nilsson J.O., Odqvist J. (2009). Study of decomposition of ferrite in a duplex stainless steel cold worked and aged at 450–500 °C. Mater. Sci. Eng. A.

[27] Wang J., Zou H., Li C., Peng Y., Qiu S., Shen B. (2008). The spinodal decomposition in 17-4PH stainless steel subjected to long-term aging at 350 °C. Mater. Charact..

[28] Malouines P., Grandemange J.M. (2011). RCC-M: Content, working approach and future evolutions. Press. Vessel. Pip. Conf..

[29] Pareige C., Emo J., Saillet S., Domain C., Pareige P. (2015). Kinetics of G-phase precipitation and spinodal decomposition in very long aged ferrite of a Mo-free duplex stainless steel. J. Nucl. Mater..

[30] Lach T.G., Collins D.A., Sang Byun T. (2021). Evolution of the role of molybdenum in duplex stainless steels during thermal aging: From enhancing spinodal decomposition to forming heterogeneous precipitates. J. Nucl. Mater..

[31] (2022). Standard Specification for Precipitation-Hardening Stainless and Heat-Resisting Steel Plate, Sheet, and Strip.

[32] Tanaka M., Choi C.S. (1972). The effects of carbon contents and Ms temperature on the hardness of martensitic Fe-Ni-C alloys. Trans. Iron Steel Inst. Jpn..

[33] Yamabe J., Sezgin J.G., Wada K. (2021). Interpretation of complex, tensile-fracture phenomena in precipitation-hardened, martensitic stainless steels, 17–4PH, in presence of hydrogen. Mater. Sci. Eng. A.

[34] Lashgari H.R., Kong C., Adabifiroozjaei E., Li S. (2020). Microstructure, post thermal treatment response, and tribological properties of 3D printed 17-4 PH stainless steel. Wear.

[35] Song Y.Y., Li X.Y., Rong L.J., Li Y.Y., Nagai T. (2014). Reversed austenite in 0Cr13Ni4Mo martensitic stainless steels. Mater. Chem. Phys..

[36] Bhambroo R., Roychowdhury S., Kain V., Raja V.S. (2013). Effect of reverted austenite on mechanical properties of precipitation hardenable 17-4 stainlesssteel. Mater. Sci. Eng. A.

[37] LeBrun T., Nakamoto T., Horikawa K., Kobayashi H. (2015). Effect of retained austenite on subsequent thermal processing and resultant mechanical properties of selective laser melted 17-4 PH stainless steel. Mater. Des..

[38] Russell K.C., Brown L.M. (1972). A dispersion strengthening model based on differing elastic moduli applied to the iron-copper system. Acta Metall..

[39] Fujii K., Fukuya K., Kasada R., Kimura A., Ohkubo T. (2015). Effects of tensile stress on Cu clustering in irradiated Fe–Cu alloy. J. Nucl. Mater..

[40] Murr L.E., Martinez E., Hernandez J., Collins S., Amato K.N., Gaytan S.M., Shindo P.W. (2012). Microstructures and Properties of 17-4 PH Stainless Steel Fabricated by Selective Laser Melting. J. Mater. Res. Technol..

[41] Chung C.Y., Tzeng Y.C. (2019). Effects of aging treatment on the precipitation behavior of ε-Cu phase and mechanical properties of metal injection molding 17-4PH stainless steel. Mater. Lett..

